# Flexural Properties, Impact Strength, and Hardness of Nanodiamond-Modified PMMA Denture Base Resin

**DOI:** 10.1155/2022/6583084

**Published:** 2022-07-09

**Authors:** Shaimaa M. Fouda, Mohammed M. Gad, Passent Ellakany, Maram A. Al Ghamdi, Soban Q. Khan, Sultan Akhtar, Mohamed S. Ali, Fahad A. Al-Harbi

**Affiliations:** ^1^Department of Substitutive Dental Sciences, College of Dentistry, Imam Abdulrahman Bin Faisal University, P.O. Box 1982, Dammam 31441, Saudi Arabia; ^2^Department of Dental Education, College of Dentistry, Imam Abdulrahman Bin Faisal University, P.O. Box 1982, Dammam 31441, Saudi Arabia; ^3^Department of Biophysics, Institute for Research and Medical Consultations, Imam Abdulrahman Bin Faisal University, P.O. Box 1982, Dammam 31441, Saudi Arabia

## Abstract

**Purpose:**

Investigate the effect of low nanodiamond (ND) addition and autoclave polymerization on the flexural strength, impact strength, and hardness of polymethylmethacrylate (PMMA) denture base.

**Methods:**

A total of 240 heat polymerized PMMA were fabricated with low ND concentrations of 0.1%, 0.25%, and 0.5%, and unmodified as control. The specimens were divided equally into group I: conventionally polymerized PMMA by water bath and group II: polymerized by the autoclave. The impact strength, flexural strength, and elastic modulus were tested using the Charpy-type impact-testing machine and three-point bending test, respectively. A scanning electron microscope (SEM) was used to analyze the fractured surfaces. Surface hardness was measured by a hardness tester with a Vickers diamond. The bonding and interaction between the PMMA and ND particles were analyzed by the Fourier-transform infrared (FTIR) spectroscope. ANOVA and post hoc Tukey test were used for data analysis (*α* = 0.05).

**Results:**

ND addition significantly increased the flexural strength of groups I and II (*p* < 0.001, *p*=0.003); it was highest (128.8 MPa) at 0.25% ND concentration for group I and at 0.1% for group II. Elastic modulus increased at 0.1% ND for both groups (*p*=0.004, *p*=0.373), but the increase was statistically significant for group I only. Impact strength showed no significant change with the addition of ND in groups I and II (*p*=0.227, *p*=0.273), as well as surface hardness in group I (*p*=0.143). Hardness decreased significantly with 0.25%ND in group II.

**Conclusion:**

The addition of ND at low concentration increased the elastic modulus and flexural strength of conventionally and autoclave polymerized denture base resin. Autoclave polymerization significantly increased the flexural strength, impact strength, and hardness of unmodified PMMA and hardness of 0.5% ND group.

## 1. Introduction

Polymethyl methacrylate (PMMA) resin is a synthetic polymer that is used commonly in the fabrication of removable prostheses [1]. PMMA is characterized by several properties that facilitates its frequent use in dentistry such as the simplicity of use and molding, resistance to biodegradation inside the oral cavity, biocompatibility, feasibility, and esthetics as well as simplicity of fabrication and repair. However, the mechanical properties of PMMA are not good enough to withstand the heavy occlusal forces as PMMA has low flexural and impact strength, dimensional instability under different thermal temperatures leading to crack propagation, and formation of microporosities and might end up with prostheses fracture [1, 2].

The common causes of denture fracture are accidental drop and continuous flexing caused by mastication [3]. Therefore, it is important that the denture base material possess adequate impact and flexural properties to reduce the probability of denture fracture [4]. The stiffness of the denture base material is presented by its elastic modulus. A high modulus of elasticity makes the denture base materials resist permanent deformation that can be caused by mastication-induced stress [5]. Hardness is another important property required in a denture base material to resist permanent surface indentation [6, 7].

Several materials (fibers and fillers) have been used for reinforcement of PMMA [8], such as metal wires, glass fibers and fillers, metal oxides, and recently nanofillers. Nanotechnology provided nanosized fillers/fibers for reinforcement of PMMA [9]. Enhancement of the acrylic resin mechanical properties depends on the concentrations and uniform distribution of the added nanoparticles, as well as the formation of the strong bond between nanoparticles and resin matrix [10, 11]. Nanodiamonds (NDs) are biocompatible, have high strength, are chemically stable, and conduct heat [12]. They also have several reactive groups (NH_2_, OH) that improve bonding with PMMA [11]. The aforementioned factors in addition to the even distribution of NDs within the resin matrix make it a good filler for PMMA.

The effect of the polymerization method on PMMA properties has been previously tested. Among these methods are visible light, autoclaves, and microwaves to speed the polymerization of PMMA without causing any deterioration in the material composition and properties [13, 14]. Autoclave polymerization depends on using the autoclave machine that is widely used in sterilization by applying steam of high pressure at a temperature of 121^o^C for 15–20 min to the contents that need to be sterilized [13, 15]. The same method is implemented on PMMA resin by applying the autoclave pressure in order to increase the degree of conversion and reduce the content of residual monomer [14]. Consequently, the decreased content of residual monomer results in improvement of PMMA properties [16].

Previous studies investigated the influence of high concentrations of NDs on the mechanical, physical, and antifungal activities of PMMA [17, 18]. However, the dual effect of ND addition and autoclave polymerization on PMMA denture base material has not been tested previously. The aim of the current study was to detect the effect of low ND and autoclave polymerization on the elastic modulus, flexural strength, hardness, and impact strength of the PMMA denture base. The first study's null hypothesis was that the tested properties will not be changed by the addition of a low concentration of ND to PMMA. The second null hypothesis was the insignificant influence of the combined effect of autoclave polymerization and low ND addition on the PMMA denture base material tested properties.

## 2. Materials and Methods

Parameters used to calculate sample size were power (80%), level of significance (5%), and confidence interval (95%); hence, the calculated sample size showed the need for 240 specimens (120 for flexural strength and hardness and 120 for impact strength) of heat polymerized PMMA to perform the present study. The specimens were equally divided into group I: conventional water bath heat polymerized and group II: autoclave polymerized PMMA. Each group included 4 subgroups of unmodified PMMA and 0.1wt%, 0.25wt%, and 0.5wt% ND added to acrylic resin powder (Major Prodotti Dentari, Moncalieri, Italy). The conventionally polymerized unmodified PMMA group served as a control.

### 2.1. PMMA/NDs Mixture Preparation

ND (Shanghai Richem International Co. Ltd, Shanghai, China) particles were heat-treated following the same method used in previous studies [17, 19]. Then, they were weighted by an electronic balance (S-234; Denver Instrument GmbH, G¨ottingen, Germany) in concentrations of 0.1%wt, 0.25%wt, and 0.5%wt of acrylic resin powder and mixed with the resin powder forming a homogenous nanocomposite mixture following the mixing method in a previous study [17].

**Figure 1:**
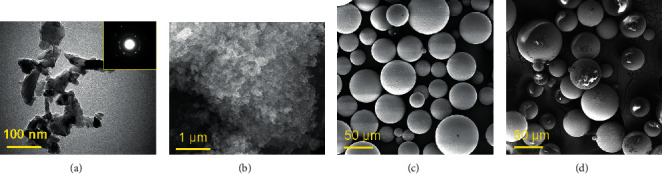
(a) TEM image of ND powder, and (inset) corresponding selected area electron diffraction (SAED) pattern showing crystalline structure; (b) SEM micrograph of ND powder; SEM micrographs of (c) pure PMMA; (d) PMMA/ND mixture.

The morphology (shape, size, and structure) of the used filler greatly affects the mechanical properties of the prepared PMMA matrix. In this regard, electron microscopy methods (scanning electron microscope (SEM) and transmission electron microscope (TEM)) are broadly utilized to visualize the shape, size, and structure of the materials. In this study, TEM (Morgagni 268, FEI) was used to reveal the morphology of pure ND powder (Figure 1(a)). Furthermore, the wide view of the pure ND powder was examined by SEM (SEM-TESCAN VEGA3 LM model) (Figure 1(b)). The physical morphology of the pure PMMA and the distribution of ND filler in the PMMA powder (PMMA/ND mixture) were viewed before heat- and autoclave processes by SEM (Figures 1(c) and 1(d)). It was seen that the shape of the ND particles was irregular, and the size varied between 20 and 200 nm within a few tens of nanometer thickness. The pure PMMA specimen showed clean spheres of PMMA with an average diameter of ∼ 50 *μ*m. The ND/PMMA mixture specimen displayed the uniform distribution of ND particles whereby ND particulates attached to PMMA spheres were clearly visible.

### 2.2. Specimens' Fabrication

Metal molds were used for specimens' fabrication with different dimensions for each test, 64 × 10 × 3.3 mm for the flexural properties test [20], 55 × 10 × 10 mm^3^ with a mid-notch for the impact strength test [21, 22], and 12 × 12 × 3 mm^3^ for the surface hardness test.

The conventional compression mold technique was used to fabricate the specimens. The molds were waxed up and invested; then, after wax elimination, packing was done as described in previous studies [13, 15, 17]. Group I were conventionally polymerized in a curing unit (KaVo Elektrotechnisches Werk GmbH, Leutkirch, Germany) at 74°C for a duration of 8 hours and then followed by 1 hour at 100°C. Group II specimens were polymerized in an autoclave (Ritter M11 UltraClave; Midmark International, Spain) at 60°C for 30 minutes followed by 130°C for 20 minutes.

The polymerized specimens were finished using a tungsten carbide bur (HM251FX-040 HP; Meisinger, Centennial, CO) followed by polishing with a mechanical polisher (Metaserve 250 grinder-polisher; Buehler, Lake Bluff, IL) for 2 minutes at 100 rpm speed in wet conditions. The accuracy of the specimens was confirmed by a digital caliper; then, they were stored in distilled water at room temperature for 48 hours.

The flexural strength and elastic modulus were calculated as explained in previous studies [13, 17] using a universal testing machine (Instron Model 8871; Instron Corp., Canton, MA), with a 5 mm/min crosshead speed and 50 kgf load applied on the specimens until fractured. The impact strength was digitally recorded using a pendulum Charpy-type impact-testing machine (Digital Charpy Izod impact tester, XJU 5.5; Jinan Hensgrand Instrument Co., Ltd., Jinan, China) [17]. Surface hardness was measured using a hardness tester (Wilson Hardness; ITW Test & Measurement, GmbH, Shanghai, China) equipped with a Vickers diamond indenter at 25-gf load for 30 seconds [15].

### 2.3. SEM Analysis

**Figure 2:**
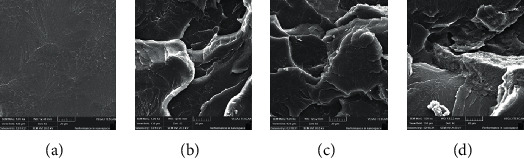
(a)–(d) Representative SEM images of group I specimens. (a) Unmodified 0%NDs, (b) 0.05% NDs-modified specimens, (c) 0.25% NDs-modified specimens, and (d) 0.5% NDs-modified specimens.

**Figure 3:**
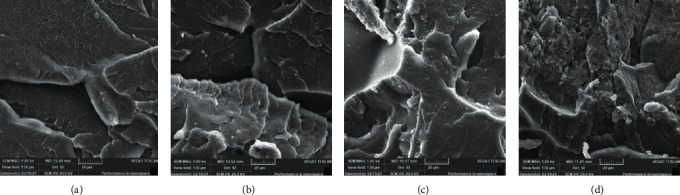
(a)–(d) Representative SEM images of group II specimens. (a) Unmodified 0%NDs, (b) 0.05% NDs-modified specimens, (c) 0.25% NDs-modified specimens, and (d) 0.5% NDs-modified specimens.

After testing, the fractured surfaces were gold coated before analysis by SEM at an accelerating voltage of 20 kV as reported in a previous study [17]. Recording of the electronic micrographs was done at magnifications of x200, x500, and x10000 to cover different features of the underneath surfaces. The illustrative micrographs were shown at a medium magnification of x1000 to display the significant surface features of specimens with different ND concentrations and polymerization methods (Figures 2 and 3).

### 2.4. Fourier-Transform Infrared (FTIR) Spectroscope

**Figure 4:**
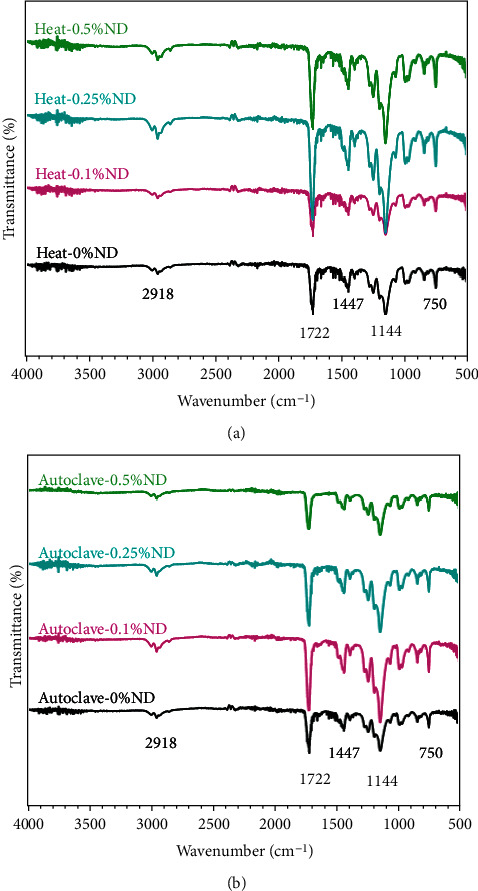
FTIR of (a) conventionally polymerized (0, 0.1, 0.25, and 0.5% ND) and autoclave specimens (0, 0.1, 0.25, and 0.5% ND) in the spectral range of 4000–500 cm^−1^.

Fourier-transform infrared spectroscopy (FTIR) is an important tool to study the chemical bonding of organic materials. Herein, FTIR (FTIR; Nicolet 6700) was utilized to study and analyze the chemical bonding between ND particles and PMMA resins, where PMMA resin was reinforced with four different concentrations of ND particles (0.1, 0.25, and 0.5 wt. % ND). The FTIR plots (4000–500 cm^−1^) are shown in Figure 4.

### 2.5. Statistical Analysis

A statistical package of social sciences (SPSS v.23) was used for data entry and analysis. Normality of the tested properties through the Shapiro Wilk test and insignificant results verified the presence of normality in the data; hence, parametric statistical tests were used for data analysis. In the descriptive analysis, mean and standard deviations were computed. In inferential statistics, One-way ANOVA was used to test the effect of variation in concentration of ND on the tested properties of groups I and II followed by the Tukey post hoc test for pairwise comparison. Furthermore, two-way ANOVA was employed to test the combined effect of concentration levels and polymerization techniques used. Two-independent-sample *t*-test was used to compare the mean difference between groups I and II within each concentration level for each tested property. *p* values less than 0.05 were considered statistically significant.

## 3. Results

**Figure 5:**
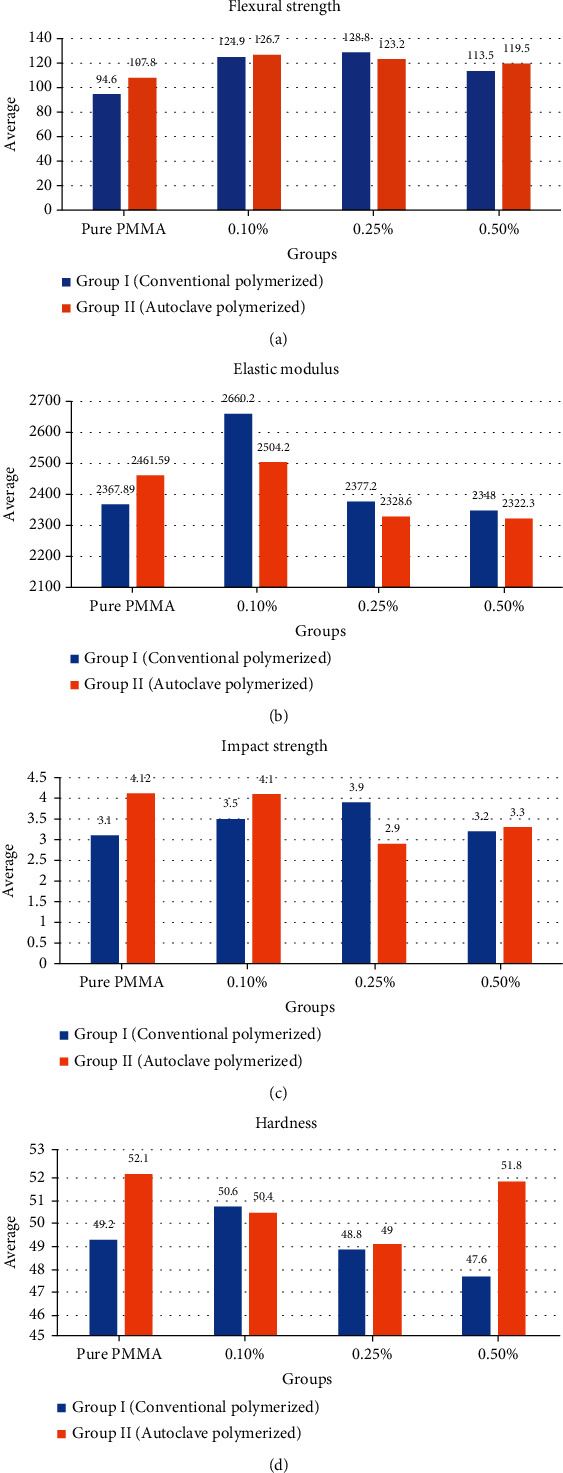
(a) Flexural strength, (b) elastic modulus, (c) impact strength, and (d) hardness of the tested materials.

The flexural strength was the highest in group I at 0.25% concentration (128.8 ± 18.4 MPa) and in group II at 0.1% (126.7 ± 9 MPa) and the lowest at unmodified PMMA for group I (94.6 ± 13 MPa) and group II (107.8 ± 3.6 MPa) (Table 1, Figure 5(a)). The concentration levels of ND had a significant effect on the flexural strength of group I (*p* < 0.001) and group II (*p*=0.003) (Table 1). In group I, the pairwise comparison showed a significant difference in unmodified PMMA versus 0.1% (*p* < 0.001), versus 0.25% (*p* < 0.001), and versus 0.5% (*p*=0.031). Similarly, in group II, the comparison of unmodified PMMA with concentration levels of 0.1% and 0.25% was statistically significant (*p*=0.002 and *p*=0.0016). The flexural strength of specimens in group II was higher than their counterparts of group I except at 0.25% ND concentration, where the flexural strength of group I was higher than that of group II. The difference was significant for unmodified autoclave polymerized PMMA versus the control group.

The elastic modulus was the highest (2660 ± 159.8 MPa, 2504 ± 415.8 MPa) at 0.1% concentration and the lowest (2348 ± 221.6 MPa, 2322 ± 183.2 MPa) at 0.5% concentration for groups I and II, respectively (Table 1, Figure 5(b)). Pairwise comparison by using the Tukey post hoc test provided significant differences for group I between unmodified PMMA versus 0.1% (*p*=0.013), 0.1% versus 0.25% (*p*=0.017), and 0.1% versus 0.5% (*p*=0.007). However, the effect was found statistically insignificant when concentration was tested with an autoclave polymerized group (*p*=0.373). Meanwhile, the elastic modulus was not significantly changed in relation to the polymerization technique.

The impact strength of group I was the highest (3.9 ± 0.7 kJ/m^2^) at 0.25% ND and lowest (3.1 ± 0.6 kJ/m^2^) at unmodified PMMA (Table 1). However, in group II, the impact strength was the highest (4.12 ± 1.2 kJ/m^2^) at unmodified PMMA and the lowest (2.9 ± 0.4 kJ/m^2^) at 0.25% ND (Figure 5(c)). Furthermore, one-way ANOVA results revealed an insignificant association between concentration levels of ND and impact strength of groups I and II (Table 2). The impact strength of group II was higher than group I with a significant difference in the unmodified PMMA group (*p*=0.028), except at 0.25% ND concentration, the impact strength of group I was significantly higher than that of group II (*p*=0.001).

In group I, surface hardness was the highest (50.6 ± -1.8VHN) with 0.1% ND and was the lowest (47 ± 2.2 VHN) with 0.5% ND. However, variation in hardness due to change in concentration was not found statistically significant (Table 1, Figure 5(d)). Contrarily, in group II, the association between hardness and ND concentration was statistically significant (*p*=0.006) (Table 2) with a maximum value (52 ± 1.1 VHN) in the unmodified PMMA group and a minimum (49 ± 1.9 VHN) at 0.25% ND (Figure 1(d)). Furthermore, the post hoc test provided pairs with significant differences were control versus 0.25% (*p*=0.009) and 0.25% versus 0.5% (*p*=0.021). Comparing surface hardness with regard to the polymerization technique showed higher values of group II compared to group I with a significant difference at unmodified PMMA (*p*=0.025) and 0.5% ND concentration (*p*=0.001) (Table 1, Figure 5(d)).

Two-way ANOVA was used to study the combined effect of ND concentration and polymerization method over tested properties. It was found that only hardness was significantly affected by the combined effect of concentration and polymerization methods (*p*=0.015) (Table 3). The surface hardness of group II was higher than group I with a significant difference for the pure group and at 0.5% ND (Table 2).

FTIR was used to analyze the bonding between filler and PMMA resins. FTIR results of the samples of group I (0, 0.1, 0.25, and 0.5% ND) and those of group II (0, 0.1, 0.25, and 0.5% ND) are shown in Figure 4. All the prepared specimens showed the characteristic bands associated with PMMA at ∼ 2918, 1447, 1387, and 1144 cm^−1^ (methyl group), ∼ 1722 cm^−1^ (ester carbonyl), ∼1239 cm^−1^ (C–O stretching modes), 750 cm^−1^ (bending of C=O), etc. It was noticed that the filler could not alter the positions of the characteristic bands of PMMA resin; only the height of some specific bands was changed or lowered/increased with the addition of filler. The maximum band intensity was observed in 0.25% ND within conventionally polymerized specimens and 0.1% ND autoclave polymerized specimens. In summary, FTIR results indicate the successful coupling of ND nanoparticles with the PMMA matrix, which could affect the mechanical properties of the final testing specimens.

Figures 2 and 3 displayed the selected SEM images of the tested specimens, i.e., the fractured surfaces of group I and II modified and unmodified specimens after performing flexural strength tests. The unmodified specimens showed a smooth surface representing a brittle fracture type for unmodified specimens (Figures 2(a) and 3(a)), unlike specimens with added ND (0.1%, 0.25%, and 0.5%ND) that showed variant topographical and morphological features with a rough surface and thicker lamellae (Figures 2(b)–2(d) and 3(b)–3(d)). With 0.25% ND addition, uneven fractured surfaces with numerous sharp step lamellae indicate ductile fracture mode, in addition to the nonappearance of clusters which indicated well dissemination of nanoparticles within the PMMA resin matrix (Figures 2(b) and 3(b)). With increasing concentration of ND addition (groups I and II specimens), especially 0.5% ND specimens, the fractured surface displayed uneven and sharp lamella but with cluster formation of ND particles within the PMMA matrices (Figures 2(d) and 3(d)).

## 4. Discussion

Restorative biomaterials are used to restore function and/or missing oral tissues [23]. With advanced nanotechnology, a new term nanobiomaterials was introduced due to the combination of biomaterials and nanoparticles which resulted in nanocomposites with improved and high performances in comparison to conventional counterparts [24].

A complete denture is a common treatment for edentulous patients whose number is still high in many countries [25]. PMMA is the most used material for the fabrication of complete and removable partials dentures and overdentures. Reinforcement of denture base material by the addition of nanofillers could improve its mechanical properties and, thus, results in better clinical performance and less liability to fracture.

The addition of NDs to PMMA significantly increased the elastic modulus and flexural strength of PMMA; therefore, the first study hypothesis was rejected. The second study hypothesis was also partly rejected since the combined effect of autoclave polymerization and ND addition to PMMA showed a significant increase in surface hardness at 0.5% ND while other properties were not significantly changed.

Denture base material is required to have sufficient flexural strength and elastic modulus to resist denture fracture or deformation under flexural forces [26]. The addition of NDs increased the flexural strength of conventionally and autoclaved polymerized groups. Bonding between the added filler and PMMA is essential to ensure the improvement of the resin's mechanical properties. In the present study, NDs were heat-treated before mixing with PMMA powder to produce surface oxide groups, to enhance their bonding with the resin matrix [17, 19]. The FTIR spectra confirmed the interaction between the surface hydroxyl groups of the ND particles and the methoxycarbonyl groups of PMMA. The FTIR spectra have also shown maximum band intensity at 0.25% ND in group I specimens and 0.1% ND in group II specimens which showed the highest flexural strength values, respectively. Moreover, the SEM analysis showed a smooth surface of unmodified PMMA in both groups indicating a brittle fracture type. With ND addition, the specimens' surface became rougher and had thicker lamellae with the absence of ND agglomeration to 0.25% concentration. Accordingly, the increase in flexural strength could have resulted from the strong bond formed between ND and PMMA resin matrix and the uniform distribution of NDs. However, the flexural strength at high ND concentration (0.5%) decreased and was the lowest among the ND modified groups for groups I and II. This could have resulted from the formation of ND clusters at high concentrations supported by the SEM analysis. In agreement with a previous study, the addition of 0.5% ND significantly increased the flexural strength of heat polymerized PMMA, but at higher ND concentration, the flexural strength was decreased [17]. Another study in line with the present results found a significant increase of autopolymerized PMMA flexural strength at ND concentrations of 0.1, 0.3, and 0.5% compared to unreinforced resin [27].

The flexural strength of specimens in group II was higher than group I counterparts except at 0.25% ND concentration. The reason for the higher flexural strength of the autoclave polymerized group could decrease the amount of residual monomer caused by autoclave elevated pressure and boiling temperature [28]. However, the increase was not statistically significant in agreement with previous studies [29–31].

The least recommended elastic modulus for denture bases is 2,000 MPa in the clinical situation [32]. The elastic modulus of conventionally and autoclave polymerized denture base resin increased with 0.1% ND but was insignificantly decreased at higher concentrations. However, the least value recorded in the present study was above 2000 MPa. The improvement in elasticity might be related to the uniform distribution of NDs incorporated in the denture resin matrix. In addition to the results of the zeta sizer, the minimal space between nanoparticles in the form of microns can minimize the polymer chain immobilization effect [33]. Similarly, Kevf et al. [34] found that the addition of reinforcement particles to provisional denture base resin produces an improved elastic modulus. Also, a previous study reported a considerable increase in elastic modulus of denture base resin among all concentrations used of SiO_2_ nanoparticles where the highest elasticity was shown in the case of the least amount of SiO_2_ NP [35]. On the same line, Albasarah et al. [36] found that the addition of nano-ZrO_2_ to denture base resin increased the elastic modulus even at a reduced thickness of denture base.

The results showed an increase in the impact strength of group I with the addition of NDs which was not statistically significant, while for group II, the addition of ND insignificantly decreased the impact strength with 0.25 and 0.5% NDs. Al-Harbi et al. [17] found a significant decrease in impact strength with the addition of NDs to heat polymerized PMMA at concentrations of 0.5, 1, and 1.5 wt %. On the contrary, Protopapa et al. [37] reported a significant increase in the impact strength with the addition of NDs to autopolymerized PMMA at concentrations of 0.1%, 0.38%, 0.5%, and 0.83%. These differences in the results of the present study might be due to the different percentages of added NDs, in addition to variation in the type of PMMA and methods of its polymerization.

The results of the current study showed that variation in hardness of group I due to change in ND concentration was not statistically significant, while the hardness of group II was significantly decreased at 0.25% ND only. On the contrary, previous studies reported increased surface hardness of PMMA with the addition of ND [27, 38, 39]. The difference in results with those of the present study may be due to differences in materials used, ND concentration, and technique of resin polymerization. However, a study did not find a significant change in denture base hardness after the addition of nanofillers in line with the present results [40].

The results showed that surface hardness was higher with autoclave polymerization. Autoclave polymerization leads to complete polymerization and a reduced amount of residual monomer resulting from the high pressure and temperature [28]. Previous studies correlated with the increased hardness of denture base material and reduced amount of residual monomer [14, 41, 42]. The surface hardness is adversely affected by the plasticizing effect of the residual monomer [14]. In agreement with the present results, previous studies reported increased hardness of denture base resin polymerized by autoclave [13–15].

According to the findings of the present study, low ND addition with both polymerization techniques could be recommended for denture base fabrication. Moreover, autoclave polymerization could be used for the fabrication of denture base resin with higher strength. It was noticed that high ND% affected the optical properties when viewed by the naked eye which could be solved by the addition of low concentrations or by the addition of high concentrations in unaesthetic areas. ND could also be added to the fitting surface of dentures by using the double-layer technique suggested by a previous study [43].

The study is limited by its in vitro nature. The specimens were not the same shape as the denture nor were subjected to conditions similar to those in the oral cavity as changes in temperature, PH, and exposure to saliva and oral flora. Other limitations are the use of only one type of PMMA and nanofiller. The effect of ND on vital tissue needs to be tested in further studies to evaluate the cytotoxicity of PMMA reinforced with ND and to determine the efficiency of its clinical application in denture fabrication. In addition, more studies are required to test the long-term effect of ND on PMMA in conditions simulating the oral cavity, as well as the effect of denture cleansers, artificial aging, and different beverages on the properties of the reinforced resin. Moreover, it is recommended to test the effect of different concentrations of ND with different resin brands and polymerization cycles.

## 5. Conclusions

The addition of ND at a low concentration significantly increased the flexural strength of conventionally and autoclave polymerized PMMA and the elastic modulus of conventionally polymerized resin. Autoclave polymerization could be used as an alternative method for polymerization of pure PMMA with a significant increase in flexural strength, impact strength, and hardness. Moreover, autoclave polymerization and the addition of 0.5%ND significantly increased the hardness of PMMA.

## Figures and Tables

**Table 1:** Means, standard deviation, and significance of tested properties.

Properties	Groups/ND%	Group I (conventional polymerized)	Group II (Autoclave polymerized)	*p* value
Flexural strength	Pure PMMA	94.6 (13)	107.8 (3.6)^a,^	0.016^*∗*^
	0.1%	124.9 (8.4)^a,b,^	126.7 (9)^b,c,^	0.685
	0.25%	128.8 (18.4)^a,c,^	123.2 (13.8)^b,d,^	0.498
	0.5%	113.5 (8.7)^b,c,^	119.5 (8.9)^a,c,d,^	0.187
	*F &p* value	*F* = 11.94, *p*=0.0001^*∗*^	*F* = 5.918, *p*=0.003^*∗*^	

Elastic modulus	Pure PMMA	2367.89 (163.1)^a,b^	2461.59 (251.3)	0.336
	0.1%	2660.2 (159.8)	2504.2 (415.8)	0.283
	0.25%	2377.2 (249.2)^a,c^	2328.6 (221.9)	0.651
	0.5%	2348 (221.6)^b,c^	2322.3 (183.2)	0.782
	*F&p* value	*F* = 5.394, *p*=0.004^*∗*^	*F* = 1.074, *p*=0.373	

Impact strength	Pure PMMA	3.1 (0.6)	4.12 (1.2)	0.028^*∗*^
	0.1%	3.5 (1.1)	4.1 (3.1)	0.534
	0.25%	3.9 (0.7)	2.9 (0.4)	0.001^*∗*^
	0.5%	3.2 (1.0)	3.3 (0.6)	0.763
	*F&p* value	*F* = 1.517, *p*=0.227	*F* = 1.352, *p*=0.273	

Hardness	Pure PMMA	49.2 (3.6)	52.1 (1.1)^a,b,^	0.025^*∗*^
	0.1%	50.6 (1.8)	50.4 (2.5)^a,c,d^	0.839
	0.25%	48.8 (3.1)	49.0 (1.9)^c^	0.885
	0.5%	47.6 (2.2)	51.8 (2.3)^b,d,^	0.001^*∗*^
	*F&p* value	*F* = 1.926, *p*=0.143	*F* = 4.822, *p*=0.006^*∗*^	

^*∗*^Statistically significant at 0.05 level of significance. Same small alphabets in each column per polymerization technique showed a statistically insignificant difference between the means.

**Table 2:** Effect of different concentration levels on tested properties of conventionally and autoclave polymerized denture base material.

	Property	Groups	Sum of Square	Df	Mean square	*F*	*P*
Group I	Flexural strength	Between groups	5655.29	3	1885.1	11.94	<0.0001^*∗*^
		Within groups	4592.16	28	164.0		
		Total	10247.4	31			
	Modulus elasticity	Between groups	660671.3	3	220223.7	5.394	0.004^*∗*^
		Within groups	1469761.2	36	40826.7		
		Total	2130432.4	39			
	Impact strength	Between groups	3.576	3	1.189	1.517	0.227
		Within groups	28.206	36	0.783		
		Total	31.772	39			
	Hardness	Between groups	44.5	3	14.852	1.926	0.143
		Within groups	277.6	36	7.711		
		Total	322.1	39			

Group II	Flexural strength	Between groups	1622.13	3	540.7	5.918	0.003^*∗*^
		Within groups	2558.3	28	91.4		
		Total	4180.4	31			
	Modulus elasticity	Between groups	256703.0	3	85567.7	1.074	0.373
		Within groups	2869471.9	36	79707.5		
		Total	3126174.9	39			
	Impact strength	Between groups	11.766	3	3.922	1.352	0.273
		Within groups	104.47	36	2.902		
		Total	116.236	39			
	Hardness	Between groups	59.453	3	19.818	4.822	0.006^*∗*^
		Within groups	147.957	36	4.11		
		Total	207.410	39			

**Table 3:** Combined effect of concentration and polymerization method on tested properties by using Two-way ANOVA.

Tested properties	Source	Type III Sum of Squares	df	Mean Square	*F*	*P*
Flexural strength	concentration	6530.688	3	2176.896	17.049	<.0001^*∗*^
	Polymerization method	236.925	1	236.925	1.856	.179
	concentration ^*∗*^ Polymerization method	746.725	3	248.908	1.949	.132
	Error	7150.479	56	127.687		
	Total	896528.785	64			

Elastic modulus	concentration	760043.856	3	253347.952	4.204	.008^*∗*^
	Polymerization method	23256.541	1	23256.541	.386	.536
	concentration ^*∗*^ Polymerization method	157330.440	3	52443.480	.870	.461
	Error	4339233.111	72	60267.127		
	Total	474277393.274	80			

Impact strength	concentration	3.582	3	1.194	.648	.587
	Polymerization method	.720	1	.720	.391	.534
	concentration ^*∗*^ Polymerization method	11.751	3	3.917	2.126	.104
	Error	132.676	72	1.843		
	Total	1142.567	80			

Hardness	concentration	37.816	3	12.605	2.133	.104
	type	61.075	1	61.075	10.334	.002^*∗*^
	Concentration ^*∗*^ Polymerization method	66.191	3	22.064	3.733	.015^*∗*^
	Error	425.541	72	5.910		
	Total	200021.030	80			

## Data Availability

The data used to support the findings of the study can be obtained from the corresponding author upon request.
